# HPV as a Molecular Hacker: Computational Exploration of HPV-Driven Changes in Host Regulatory Networks

**DOI:** 10.3390/v17091166

**Published:** 2025-08-27

**Authors:** Massimiliano Chetta, Alessandra Rosati, Nenad Bukvic

**Affiliations:** 1 Azienda Ospedaliera di rilievo nazionale “A. Cardarelli Hospital’s” Laboratory of Medical Genetics and Genomics, 80131 Naples, Italy; 2 Department of Medicine, Surgery and Dentistry “Schola Medica Salernitana”, University of Salerno, 84081 Baronissi, Italy; 3 U.O.C Genetica Medica, Azienda Ospedaliero—Universitaria Consorziale Policlinico di Bari, 70124 Bari, Italy

**Keywords:** HPV (Human Papillomavirus), transcription factor binding sites (TFBSs), transcription factors (TFs)

## Abstract

Human Papillomavirus (HPV), particularly high-risk strains such as HPV16 and HPV18, is a leading cause of cervical cancer and a significant risk factor for several other epithelial malignancies. While the oncogenic mechanisms of viral proteins E6 and E7 are well characterized, the broader effects of HPV infection on host transcriptional regulation remain less clearly defined. This study explores the hypothesis that conserved genomic motifs within the HPV genome may act as molecular decoys, sequestering human transcription factors (TFs) and thereby disrupting normal gene regulation in host cells. Such interactions could contribute to oncogenesis by altering the transcriptional landscape and promoting malignant transformation.We conducted a computational analysis of the genomes of high-risk HPV types using MEME-ChIP for de novo motif discovery, followed by Tomtom for identifying matching human TFs. Protein–protein interactions among the predicted TFs were examined using STRING, and biological pathway enrichment was performed with Enrichr. The analysis identified conserved viral motifs with the potential to interact with host transcription factors (TFs), notably those from the FOX, HOX, and NFAT families, as well as various zinc finger proteins. Among these, SMARCA1, DUX4, and CDX1 were not previously associated with HPV-driven cell transformation. Pathway enrichment analysis revealed involvement in several key biological processes, including modulation of Wnt signaling pathways, transcriptional misregulation associated with cancer, and chromatin remodeling. These findings highlight the multifaceted strategies by which HPV may influence host cellular functions and contribute to pathogenesis. In this context, the study underscores the power of in silico approaches for elucidating viral–host interactions and reveals promising therapeutic targets in computationally predicted regulatory network changes.

## 1. Introduction

Human Papillomavirus (HPV) is a double-stranded DNA virus belonging to the *Papillomaviridae* family. It includes more than 200 types, some of which are known for their role in the development of cervical cancer, other anogenital cancers, and oropharyngeal carcinoma. Although HPVs were identified prior to the 1980s, it was during this decade that their etiological role in oncogenesis, particularly cervical carcinogenesis, was definitively established. Subsequently, HPV types were stratified into high-risk and low-risk categories based on their oncogenic potential. Due to its extensive prevalence worldwide, HPV represents not only one of the most common sexually transmitted infections (STI) but is, in fact, the most prevalent STI globally, with significant implications for public health [1].

HPV infection is often asymptomatic, but in symptomatic cases, it can cause benign lesions (such as condylomas) or, in the case of high-risk strains, lead to neoplasia. In particular, HPV16 and HPV18 strains are responsible for the majority of cervical cancer cases. Scientific interest in this virus has grown significantly due to its etiological role in several cancer pathologies. HPV is primarily transmitted through sexual contact but can also spread through skin-to-skin contact or contaminated surfaces. Persistent infection with high-risk strains is associated with an increased risk of developing dysplasia and carcinomas. Screening programs, such as the Pap smear and the HPV-DNA test, are essential for early diagnosis and prevention of cervical cancer [2].

The viral genome consists of approximately 8000 base pairs and includes early (E) and late (L) genes. The early genes (*E1*, *E2*, *E4*, *E5*, *E6*, and *E7*) are involved in viral replication and oncogenesis, while the late genes (*L1* and *L2*) encode the structural proteins of the virus [2]. The interaction between HPV and host cellular mechanisms is crucial for the virus life cycle and its pathogenic influence on the infected cells. HPV hijacks the host transcriptional machinery to promote its own replication and persistence. For example, the viral oncoproteins E6 and E7 can inactivate the cancer suppressor proteins p53 and Rb, respectively, disrupting cell cycle regulation and promoting uncontrolled cell proliferation [3].

In addition to the well-characterized roles of the viral oncoproteins E6 and E7, HPV replication and transcription are intricately regulated through interactions with host chromatin organizers and transcriptional regulators. A central factor in this process is CTCF (CCCTC-binding factor), a key architectural protein that binds specific sites within the HPV genome. CTCF plays a critical role in regulating viral transcription by promoting chromatin looping, which modulates promoter-enhancer interactions and alternative splicing, particularly within the early region of HPV18 [4]. Furthermore, it was demonstrated that disruption of CTCF binding in HPV16 results in aberrant activation of viral oncogene expression during differentiation and loss of genome looping, highlighting the significance of CTCF binding in preserving the appropriate temporal control of viral transcription [5]. In parallel, HPV exploits SMC1 (Structural Maintenance of Chromosomes 1), a component of the cohesin complex, to tether the viral genome to host mitotic chromosomes, thereby ensuring episomal persistence during cell division. SMC1 colocalizes with CTCF at viral chromatin domains, suggesting a coordinated mechanism through which HPV maintains its episomal status while synchronizing replication with host cell cycle progression [5].

Later, a more comprehensive molecular framework was presented, demonstrating how HPV’s replication mechanism incorporates additional chromatin-associated proteins. Histone modifiers such as histone acetyltransferases (HATs) and deacetylases (HDACs), as well as chromatin remodelers that influence nucleosome accessibility and placement, are known to interact with HPV in addition to CTCF and cohesins. These relationships affect the epigenetic environment of infected cells as well as transcription and viral genome maintenance, enabling a dynamic and differentiation-dependent control of viral gene expression [6].

Beyond the replication cycle, HPV-driven oncogenesis is further potentiated by the abduction of host transcription factors that govern cell proliferation, immune response, and survival. Members of the STAT (Signal Transducer and Activator of Transcription) family, particularly STAT3, have been found constitutively activated in HPV-positive cancers and contribute to both immune evasion and upregulation of viral oncogenes [7]. Similarly, the AP-1 (Activator Protein-1) transcription factor complex, composed of JUN and FOS family proteins, is induced by HPV infection and directly binds the long control region (LCR) of the viral genome, enhancing the transcription of E6 and E7 and promoting cellular transformation [8]. Recent studies have also shown that the Hippo pathway effectors TAZ (WWTR1) and YAP (Yes-associated protein) are important in HPV-mediated carcinogenesis. Following recruitment by HPV regulatory domains, YAP/TAZ activity collaborates with viral components to sustain oncogenic signals, which promote cell proliferation. Additionally, HPV immortalizes normal human keratinocytes primarily through the action of their E6 and E7 oncoproteins and independently from the life cycle [9,10].

Together, these findings highlight the multifaceted strategies employed by HPV to manipulate the host epigenetic and transcriptional machinery, enabling both persistent infection and malignant progression.

We present a computational analysis designed to identify potential interactions between human transcription factors (TFs) and the transcription factor binding sites (TFBSs) that are shared among the major high-risk oncogenic strains of human papillomavirus (HPV) linked to cancer development. Our hypothesis is that the sequestration of these transcription factors by viral TFBSs may disrupt normal host gene expression, resulting in effects similar to a loss of function. This in silico approach, previously applied to other viruses such as SARS-CoV-2 and arboviruses, has already proven successful in revealing how viral RNAs can sequester host RNA-binding proteins and TFs, thereby interfering with cellular processes, modulating immune responses, and in some cases opening perspectives for innovative therapeutic strategies, including vaccine development. Applied to HPV, this strategy could provide valuable insights into the mechanisms driving pathogenesis and its associated complications [11,12].

## 2. Materials and Methods

The analysis pipeline consists of four main stages and is built using various openly available bioinformatics tools. The in silico approach was applied to the genome sequences of the major HPV strains associated with cancer (Table 1) to evaluate the presence of conserved motifs capable of interacting with host proteins (https://pave.niaid.nih.gov/explore/reference_genomes/human_genomes (accessed on 1 February 2025)).

MEME-ChIP, a tool from the MEME Suite (http://meme-suite.org/tools/meme-chip, (accessed on 11 February 2025)), was used to analyze the genomic sequences of the main HPV strains. MEME-ChIP performs comprehensive motif analysis, including de novo motif discovery, on large sets of DNA sequences identified in ChIP-seq or CLIP-seq experiments. It integrates multiple tools, such as MEME, DREME, CentriMo, and FIMO, to identify enriched DNA-binding motifs (TFBSs) and their positional bias, making it suitable for uncovering regulatory elements in viral genomes. The statistical significance of the discovered motifs is assessed using the E-value, with lower values indicating higher confidence; in our analysis, the top motifs showed extremely significant E-values ranging from 5.7 × 10^−128^ to 6.7 × 10^−127^ [13].

**Figure 1:**
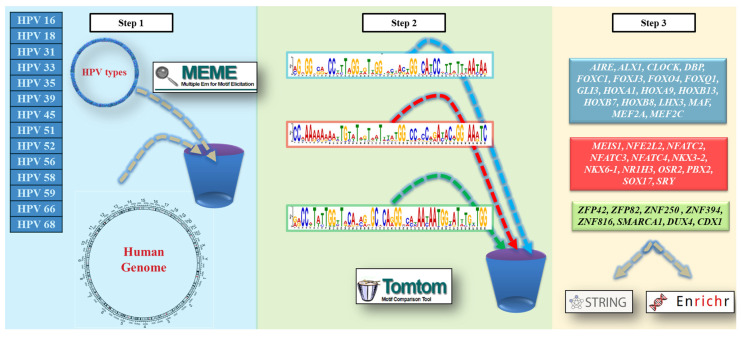
Bioinformatics pipeline used to detect human TFs associated with high-risk HPV strains. The workflow is divided into three main steps. Step 1: Genomic sequences from 15 high-risk HPV types (HPV16, HPV18, HPV31, HPV33, HPV35, HPV39, HPV45, HPV51, HPV52, HPV56, HPV58, HPV59, HPV66, and HPV68) were collected and analyzed using the MEME-ChIP tool from the MEME Suite. This tool performs de novo motif discovery to identify overrepresented DNA motifs across the viral sequences. The human genome was used as a reference background to filter out nonspecific motifs. HPV genome sequences are analyzed by MEME to generate motifs, which are carried forward as shown by the dashed blue arrows. Step 2: The top three most statistically significant motifs (based on E-values ranging from 5.7 × 10^−128^ to 6.7 × 10^−127^) were selected and visualized as sequence logos. These motifs were then compared to known transcription factor binding site (TFBS) databases using Tomtom, a motif comparison tool. Each motif is color-coded (light blue, red, or green) and connected via dashed lines to its corresponding TFs, allowing direct visual mapping between motif discovery and TF annotation. Step 3: The transcription factors predicted to bind each motif were grouped and color-coded accordingly (light blue, red, and green boxes) to match the motifs in Step 2. These TFs were further analyzed using Enrichr for functional enrichment (e.g., biological processes, molecular functions, pathways) and STRING for protein-protein interaction network analysis, as indicated by the arrows. This integrative approach helps to reveal potential host regulatory factors hijacked by HPV and their involvement in cellular pathways relevant to viral infection and carcinogenesis.

All identified motifs were used as queries for Tomtom (http://meme-suite.org/doc/tomtom.html (accessed on 20 February 2025)), another component of the MEME Suite that compares discovered motifs to a curated, non-redundant database of known DNA-binding protein motifs experimentally validated in the human genome. Tomtom uses a statistical framework based on the Benjamini-Hochberg method to calculate q-values, which represent the minimum false discovery rate (FDR) at which the observed similarity would be deemed significant. This allowed us to generate a list of TFs recognizing the conserved domains found across all viral genome motifs (Figure 1) [14].

Next, we used STRING v11.5 (https://string-db.org/ (accessed on 20 February 2025)), a database and web resource dedicated to protein–protein interaction (PPI) networks. STRING aggregates known and predicted associations derived from multiple sources, including experimental data, computational prediction methods, and public text collections. The tool was used to identify the strongest associations among the set of predicted TFs using a guilt-by-association approach, providing insight into their functional relationships within the host [15].

Finally, the entire list of TFs was used as input for Enrichr (https://maayanlab.cloud/Enrichr/ (accessed on 20 February 2025)), a web-based application that integrates multiple enrichment analysis methods and offers interactive visualizations of the results using the Data Driven Documents (D3) JavaScript library. Enrichr supports a wide range of biological databases and provides detailed graphical summaries of enriched terms, helping to interpret the collective functions of the input TF list and to explore their roles in host gene regulatory networks (Figure 1) [16].

**Table 1:** High-risk HPV types included in the analysis, along with their prevalence in cancers, cancer localization, clinical notes, genome access information, and the target of prophylactic vaccinations. (https://pave.niaid.nih.gov/explore/reference_genomes/human_genomes (accessed on 20 February 2025)) [17].

HPV Type	Prevalence in Cancers	Cancer Localization	Clinical Notes
**HPV 16**	~60% of cervical cancers ~70% of oropharyngeal cancers Also prevalent in anal, penile, vulvar, and vaginal cancers	Cervix, oropharynx, anus, penis, vulva, vagina	Highly oncogenic; persistent infection often involves integration into the host genome; primary target of prophylactic vaccines, including Gardasil 9
**HPV 18**	~10–15% of cervical cancers Significant prevalence in oropharyngeal and anal cancers	Cervix, vagina, anus, oropharynx	Strongly associated with glandular carcinomas (adenocarcinomas); included in all major HPV vaccines (Gardasil, Gardasil 9, Cervarix)
**HPV 31**	2–5% of cervical cancers	Cervix, anus, penis	Common in high-grade precancerous lesions (CIN2/3); included in Gardasil 9 vaccine
**HPV 33**	2–4% of cervical cancers	Cervix, vulva	Frequent in precancerous lesions; covered by Gardasil 9 vaccine
**HPV 35**	<2% of cervical cancers	Cervix, vagina	Detected in advanced cervical neoplasms; classified as high-risk
**HPV 39**	<2% of cervical cancers	Cervix, anus	Moderate oncogenic potential; high-risk type
**HPV 45**	~5% of cervical cancers	Cervix, vagina	Strong association with adenocarcinomas; included in Gardasil 9 vaccine
**HPV 51**	~1–2% of cervical cancers	Cervix	Rare but oncogenic; classified as high-risk
**HPV 52**	~2–3% of cervical cancers	Cervix, anus	Included in Gardasil 9 vaccine; prevalent in East Asia
**HPV 56**	<1% of cervical cancers	Cervix	Lower relative oncogenic risk; classified as probable high-risk
**HPV 58**	~2–4% of cervical cancers	Cervix, vulva	Common in East Asian populations; included in Gardasil 9 vaccine
**HPV 59**	<1% of cervical cancers	Cervix	Rare but classified as high-risk
**HPV 66**	Rarely isolated in cancers	Cervix	Frequently found in co-infections with other HPV types; probable high-risk
**HPV 68**	~1% of cervical cancers	Cervix, oropharynx	May integrate into the host genome; classified as high-risk; included in Gardasil 9 vaccine

## 3. Results

The oncogenic transformation driven by high-risk human papillomavirus (HPV), particularly HPV16 and HPV18, represents a multifactorial process that extends beyond the classical disruption of cancer suppressors such as p53 and pRb. While the E6 and E7 oncoproteins mediate these canonical interactions (via E6-AP–dependent p53 degradation and E2F release through pRb inactivation), they also orchestrate a pervasive transcriptional reprogramming of host cellular networks [18]. Using a computational pipeline that integrates multiscale data with interaction-based inference, we identified several TF families as potential targets for sequestration by viral genomes. This sequestration could reduce the availability of TFs for normal host gene regulation, thereby altering host gene expression profiles and potentially contributing to viral pathogenesis and immune evasion. This approach provided a more comprehensive and systematic identification of TFs involved in this process, which was previously challenging to delineate. Importantly, we also identified a subset of transcriptional regulators not previously linked to HPV-driven oncogenesis, suggesting they play unrecognized but potentially critical roles in the reprogrammed cellular state (Table 2).

Among the more abundant TFs identified are proteins of the Forkhead Box family (FOX): FOXO4, which is crucial in apoptosis and DNA damage response, is rendered functionally inactive due to the degradation of its primary modulator, p53 [19]. FOXC1 and FOXQ1, typically involved in embryonic development and cell migration, become deregulated as a result of chromatin instability induced by E7, promoting mesenchymal and invasive traits [20,21]. Similarly, FOXJ3, which regulates phenotypic plasticity, may undergo epigenetic alterations that impact epithelial-to-mesenchymal transition (EMT) [22].

The Homeobox (HOX) family represents a critical target in the context of viral infections with epithelial tropism, such as HPV. Members including HOXA1, HOXA9, HOXB7, HOXB8, and HOXB13 (key regulators of cellular identity and differentiation along the anteroposterior axis) are frequently dysregulated following the integration of viral DNA into the host genome. This is particularly relevant in basal epithelial cells, where these TFs are actively expressed and where HPV initiates its life cycle by replicating in the host cell nucleus in a differentiation-dependent manner. The resulting disruption of HOX gene expression contributes to a loss of cellular differentiation and promotes uncontrolled proliferation, often leading to aggressive and de-differentiated phenotypes [23]. This oncogenic potential is further amplified by the dysregulation of HOX-interacting cofactors such as MEIS1, PBX2, and LHX3, which normally cooperate in forming transcriptionally active HOX complexes [24]. Together, these alterations foster a transcriptional environment conducive to malignant transformation in epithelial tissues.

Within the immune and inflammatory regulatory axis, members of the NFAT (nuclear factor of activated T-cells) transcription factor family (particularly NFATC2, NFATC3, and NFATC4) undergo functional repression due to disrupted intracellular calcium signaling observed in HPV-infected epithelial cells. Since NFATs require calcium-dependent dephosphorylation for nuclear translocation, HPV-mediated signaling interference attenuates their transcriptional activity, compromising T cell activation and facilitating viral immune evasion [25]. This is especially relevant in the basal cells of the stratified epithelium, where HPV infection is initially established and where these transcription factors play essential roles in immune modulation. In parallel, the HPV E7 oncoprotein has been shown to associate with histone deacetylase (HDAC) complexes, promoting aberrant chromatin remodeling and transcriptional repression of genes associated with differentiation [26]. This interaction significantly affects the MEF2 (myocyte enhancer factor 2) family (especially MEF2A and MEF2C), leading to the suppression of epithelial differentiation pathways and the concurrent activation of proliferation-associated and oncogenic transcriptional programs [27]. This mechanism contributes to the virus’s ability to exploit the differentiation-dependent replication cycle of epithelial cells, maintaining replication competence in the nucleus of partially differentiated cells.

Moreover, persistent expression of E7 induces oxidative stress in infected cells, leading to the aberrant activation of NFE2L2 (also known as Nrf2), a key regulator of antioxidant responses. Under chronic stress conditions, Nrf2 becomes stabilized and accumulates in the nucleus, where it activates a transcriptional program that enhances cytoprotective and anti-apoptotic gene expression. This not only supports the survival of infected cells but also facilitates viral persistence and progression toward malignancy by promoting an environment resistant to immune-mediated clearance and apoptosis [28].

Beyond the well-characterized transcriptional regulators previously associated with HPV-driven oncogenesis, our analysis identified a cohort of TFs not traditionally linked to HPV-mediated transformation, thereby significantly broadening the scope of the virus’s regulatory disruption. This is particularly relevant given HPV’s epithelial tropism and its unique life cycle, which is tightly linked to the differentiation gradient of stratified squamous epithelium. Interestingly, GLI3, a key effector of the Hedgehog signaling pathway, was found to be downregulated in HPV-infected tissues, suggesting interference with cellular programs involved in tissue patterning, stem cell maintenance, and epithelial homeostasis. Since Hedgehog signaling is known to regulate epithelial regeneration and basal cell proliferation, its disruption could enhance the de-differentiation and proliferative features observed in HPV-related lesions [29].

Likewise, inhibition of SRY, a master regulator of SOX family members and sex-specific gene expression, was observed across multiple datasets. While SRY itself is not consistently associated with the tumor immune microenvironment, its expression changes may reflect indirect involvement in DNA repair or immune evasion processes, potentially mediated via epigenetic modifications or interaction with other transcriptional regulators. Notably, SOX family members are known to influence stemness and epithelial-to-mesenchymal transition, both of which are critical in viral persistence and cancer progression [30].

In parallel, CLOCK, a central regulator of circadian rhythms, appears to be vulnerable to HPV-mediated interference. CLOCK forms the core circadian complex with BMAL1, governing the synchronization of cellular metabolism, immune surveillance, and DNA repair [31]. Although HPV oncoproteins E6 and E7 are best known for inactivating p53 and pRb, emerging evidence indicates that they may also affect circadian machinery. Viruses, including HPV, have been shown to exploit circadian vulnerabilities to optimize replication and immune evasion, particularly by targeting transcriptional oscillators like CLOCK and BMAL1 [31]. This disruption of host circadian rhythms is not merely a secondary molecular event; it has direct consequences for tumorigenesis, immune modulation, and therapeutic responsiveness. Disrupted circadian regulation can desynchronize metabolic and immune homeostasis in epithelial tissues, creating a permissive environment for HPV persistence, immune evasion, and malignant transformation [32,33].

In addition, members of the zinc finger (ZNF) transcription factor family, including ZNF250, ZNF394, ZNF816, ZFP42, and ZFP82, exhibited marked transcriptional deregulation in HPV-infected epithelial tissues, suggesting a widespread disruption of DNA-binding fidelity and potential compromise of transcriptional specificity. This is particularly relevant in the context of HPV’s strong epithelial tropism, where infection is initiated in the basal cells of stratified squamous epithelium. These basal cells serve as reservoirs for HPV replication and transformation, given their proliferative capacity and expression of key TFs, including zinc finger proteins [34]. The virus replicates in the nucleus of keratinocytes that are differentiating, and this transcriptional regulation of the host is closely related to this differentiation-dependent replication. In this context, the activity of viral oncoproteins E6 and E7, which are known to disrupt chromatin-modifying complexes and transcriptional regulators, as well as deeper epigenomic instability brought on by viral integration into the host genome, may be reflected in the dysregulation of ZNF proteins. Zinc finger proteins, many of which act as sequence-specific repressors or activators, are critical for maintaining cell identity and transcriptional balance. Their altered expression may thus contribute to the transcriptional reprogramming observed during HPV-mediated carcinogenesis, facilitating immune evasion, loss of epithelial differentiation, and enhanced proliferative signaling. Such molecular alterations are consistent with a broader oncogenic strategy employed by HPV, where the modulation of host transcriptional networks, including those governed by ZNF TFs, supports viral persistence, transformation, and progression toward malignancy [34].

The presented data revealed also alterations in a group of TFs involved in lineage specification, immune regulation, and epithelial tissue identity, including MAF, SOX17, NR1H3, NKX3.2, NKX6.1, ALX1, OSR2, and AIRE. These TFs are integral to the establishment of morphogenetic patterning, maintenance of tissue-specific functions, and immune homeostasis. Their dysregulation in HPV-infected tissues, particularly those of epithelial origin, may reflect a broader viral strategy to disrupt basal epithelial transcriptional programs to favor persistent infection, immune escape, and carcinogenesis.

MAF is a critical regulator of terminal differentiation in immune and epithelial lineages, especially Th2 cells and anti-inflammatory pathways. Its repression in HPV-positive cells may impair epithelial maturation and promote an inflammatory environment that supports viral persistence [35]. SOX17, a member of the SRY-box family, is essential for endodermal lineage commitment and functions as a tumor suppressor by repressing Wnt/β-catenin signaling. Its downregulation may remove barriers to uncontrolled proliferation and dedifferentiation in HPV-infected basal keratinocytes, where viral replication is initiated [36].

NR1H3 (LXRα), a nuclear receptor involved in lipid metabolism and immune regulation, modulates macrophage activation and promotes anti-inflammatory responses. HPV-mediated suppression of NR1H3 may thus impair innate immune surveillance in infected epithelia [37]. Similarly, NKX3.2 and NKX6.1, members of the NK homeobox family, are key regulators of developmental patterning, particularly in mesodermal and pancreatic tissues, and their loss is associated with increased cellular plasticity and tumor aggressiveness [38,39]. The transcriptional suppression of OSR2 and ALX1, which promote urogenital and craniofacial morphogenesis, in HPV-transformed cells may indicate a dedifferentiation signature driven by epigenetic dysregulation of EMT invasion and transition [40,41]. AIRE (autoimmune regulator) is a master transcription factor that governs central immune tolerance by promoting the ectopic expression of tissue-restricted antigens (TRAs) in medullary thymic epithelial cells (mTECs). This process is essential for the deletion of autoreactive T cells during thymic development, ensuring the establishment of peripheral immune tolerance. In the context of HPV infection (particularly HPV16, which exhibits strong epithelial tropism and infects basal keratinocytes), there is growing evidence that the virus can interfere with immune regulatory networks both locally and systemically [42]. Notably, the HPV16 E7 oncoprotein has been shown to be ectopically expressed in the thymic epithelium, where it disrupts normal T cell development and impairs the physiological involution of the thymus with age. This sustained disruption of thymic architecture may lead to altered negative selection of autoreactive T cells, compromising immune surveillance against HPV-infected cells.

Moreover, downregulation of AIRE expression in HPV-positive lesions may further hinder the immune system’s ability to recognize and respond to infected epithelial cells. By suppressing the expression of TRAs in both central (thymic) and potentially peripheral contexts, HPV may facilitate immunological escape and establish persistent infection, a critical step in its oncogenic progression. These immunomodulatory effects are particularly relevant in stratified squamous epithelia, where HPV replicates in a differentiation-dependent manner and evades immune detection despite ongoing cellular proliferation in the basal and suprabasal layers [42,43]. The coordinated repression of this diverse TF cohort suggests that HPV infection orchestrates a collapse of transcriptional programs safeguarding epithelial differentiation, tissue identity, and immune self-recognition. This could drive cellular dedifferentiation, impair lineage-specific gene expression, and dismantle immune tolerance, ultimately supporting persistent infection and malignant progression.

**Figure 2:**
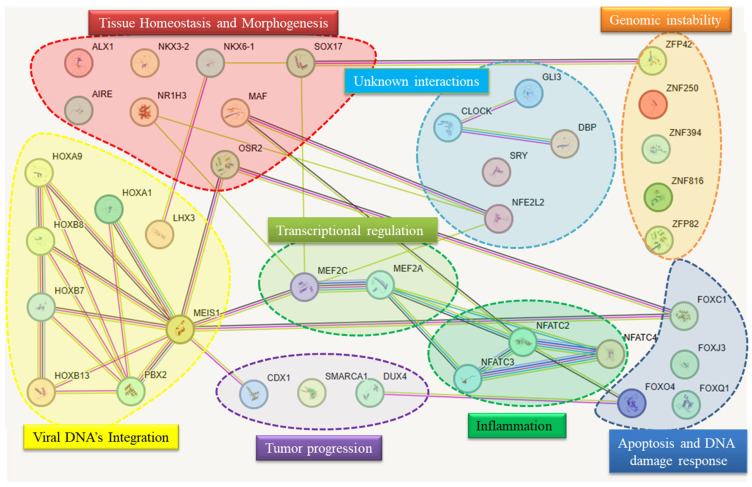
Identified transcription factor PPIs (Protein-protein interactions). The diagram displays functional and physical associations among transcription factors predicted to bind motifs discovered in high-risk human papillomavirus genomes. The network was constructed using the STRING database, which integrates evidence from experimental data, computational prediction, and public text collections to infer protein associations. Each node corresponds to a human TF, while colored lines represent different types of functional or physical associations between transcription factors. Each color corresponds to a specific predicted interaction category (e.g., co-expression, co-localization, experimental/biochemical evidence, text-mining associations, database-curated links, or protein homology). Thicker lines indicate stronger confidence scores or higher evidence support for the predicted interaction, whereas thinner lines represent weaker or less-supported associations. The TFs are color-coded and grouped into functional modules, each encircled and labeled based on their predominant biological roles. The network highlights several central hubs, such as MEIS1, MEF2A, MEF2C, and NFATC2, which appear as convergence points between multiple biological processes. Their connectivity suggests a potential regulatory bottleneck that HPV may exploit to manipulate host transcriptional programs, favoring viral persistence, immune evasion, and oncogenesis.

As shown in Figure 2, three additional TFs (SMARCA1, DUX4, and CDX1) were identified as potential direct or indirect targets of the HPV genome and were significantly associated with tumor progression in mammalian systems.

SMARCA1, a core component of the SWI/SNF chromatin remodeling complex, was significantly downregulated following HPV E7 expression. Given SMARCA1’s role in maintaining chromatin accessibility and genomic stability, its suppression may promote epigenetic drift, increased mutation rates, and loss of transcriptional fidelity [44]. DUX4, a double homeobox TF with key functions in early embryogenesis and interferon response activation, was also found to be profoundly silenced in HPV-positive environments. This suppression likely contributes to the attenuation of host antiviral pathways and supports an immunosuppressive state [45]. CDX1, a caudal-type homeobox gene involved in epithelial patterning and a known repressor of Wnt signaling, was also consistently repressed in HPV-transformed cells. Loss of CDX1 expression may contribute to epithelial identity erosion and activation of EMT programs, central to metastasis and tumor progression [46].

### Enrichr-Based Pathway Enrichment Analysis

To better contextualize the scope and functional significance of these TF connections with HPV’s genomes, we performed an enrichment analysis using Enrichr (https://maayanlab.cloud/Enrichr/ (accessed on 10 March 2025)), integrating TF targets across multiple pathway databases, including KEGG, Reactome, WikiPathways, and BioPlanet. This analysis revealed several canonical pathways enriched in TF targets but also highlighted a set of non-canonical and underexplored signaling cascades with potential relevance to HPV-mediated oncogenesis [47].

Notably, the cGMP-PKG signaling pathway emerged as significantly enriched, reflecting a potential point of viral interference through HPV16 E6-induced activation of ST6GAL1, a sialyltransferase involved in glycosylation and signal modulation. ST6GAL1 is upregulated in HPV-infected epithelial cells and has been shown to trigger downstream cGMP–PKG signaling, thereby promoting cell survival, migration, and proliferation in cervical epithelial tissues. Given HPV’s epithelial tropism and its life cycle dependency on basal keratinocytes, which support viral replication in the nucleus as they undergo differentiation, modulation of such signaling pathways may represent a broader viral strategy to ensure persistence and oncogenic transformation [48]. The E6 and E7 oncoproteins of high-risk HPV types like HPV16 are known to rewire host transcriptional programs to suppress apoptosis and bypass differentiation checkpoints. In this context, upregulation of ST6GAL1 may create a microenvironment conducive to viral replication, immune evasion, and cellular reprogramming [49].

Furthermore, perturbation of the cGMP-PKG pathway may interfere with the activity of transcription factors such as NFE2L2 (NRF2) and FOXO4, which play essential roles in oxidative stress response and cell cycle regulation. HPV-mediated suppression of their pro-apoptotic functions could facilitate immune escape and support long-term viral persistence [50,51].

Surprisingly, the oxytocin signaling pathway, traditionally associated with neuroendocrine regulation, was significantly enriched through its association with MEF2 family transcription factors. This finding suggests that HPV may interfere with the anti-proliferative and differentiation-promoting effects that this pathway exerts in epithelial tissues. MEF2 proteins are not only involved in muscle and neuronal differentiation but also play critical roles in epithelial cell fate and apoptosis. In HPV-infected epithelial cells, particularly within the basal layers where the virus initiates its replication, dysregulation of MEF2 may reflect an adaptive mechanism employed by the virus to subvert host differentiation programs. Converging evidence indicates that MEF2 activity is modulated downstream of HPV oncoproteins via histone modifications, including altered acetylation and chromatin accessibility, potentially mediated by viral interference with histone deacetylases (HDACs) and p300/CBP coactivators. These epigenetic disruptions may suppress MEF2-driven transcription of growth-inhibitory genes, thereby promoting cell survival and proliferation, hallmarks of HPV-mediated transformation [52].

The Wnt signaling pathway also showed robust enrichment, aligning with the repression of CDX1 (a known antagonist of Wnt) and the activation of forkhead box (FOX) transcription factors, particularly FOXA1 and FOXO4. This transcriptional shift reinforces HPV’s ability to EMT. Given that HPV infects basal epithelial cells, where Wnt signaling regulates self-renewal and differentiation, the virus may exploit this pathway to disrupt epithelial homeostasis and sustain a proliferative, undifferentiated state. Moreover, by repressing CDX1 and modulating FOX TF activity, HPV orchestrates a transcriptional landscape that promotes cellular plasticity, immune evasion, and long-term persistence in host tissues [53,54].

The KEGG category “Transcriptional Misregulation in Cancer” emerged as one of the top-ranked enriched pathways, reflecting the extensive involvement of deregulated TF families, including HOX, FOX, SOX, and ZNF, in HPV-associated carcinogenesis. These TF families are critical for epithelial lineage commitment, differentiation, and immune regulation, and their perturbation suggests that HPV reprograms host transcriptional networks in basal epithelial cells, thereby supporting viral replication, suppressing apoptosis, and promoting malignant transformation [55,56,57].

Among the additional significantly enriched pathways, the “Fluid Shear Stress and Atherosclerosis” pathway offers compelling insights into the broader regulatory disruptions induced by HPV infection. While this pathway is traditionally explored in the context of cardiovascular biology, emerging evidence highlights its involvement in cellular responses to oxidative and mechanical stress, which are also relevant in stratified epithelial tissues. Notably, this pathway features key transcription factors such as NFE2L2 and FOXO4, both of which are central regulators of redox homeostasis, apoptosis, and cellular adaptation to biomechanical stimuli [58,59]. NFE2L2 orchestrates antioxidant defenses by upregulating cytoprotective and detoxifying genes, whereas FOXO4 regulates apoptosis, DNA damage responses, and resistance to oxidative insults. In the context of HPV’s epithelial tropism, particularly its replication within basal epithelial cells, modulation of these mechano-sensitive TFs suggests a strategic viral mechanism to suppress apoptosis and promote cellular survival under stress conditions [60]. Our findings suggest that the removal of host TFs may disrupt these stress-adaptive pathways, creating a tumor-permissive environment by promoting oxidative resistance and suppressing pro-apoptotic signaling. The potential of HPV to take advantage of non-canonical signaling circuits to support viral persistence and neoplastic transformation is reflected in this previously ignored axis of host-virus interaction.

The “Cellular Senescence” pathway, a critical safeguard against malignant transformation, was notably impaired in HPV-infected epithelial cells, as indicated by the downregulation of key transcription factors FOXO4 and DUX4. Both factors play essential roles in initiating oncogene-induced senescence (OIS) and halting abnormal cell proliferation. FOXO4, in particular, helps maintain genomic stability and prevents tumorigenesis by promoting cell cycle arrest in response to stress signals, while DUX4 has been implicated in early embryonic transcriptional programs and antiviral defense, including the activation of interferon-stimulated genes [61]. Their silencing in the context of HPV replication within the basal layer of stratified epithelia suggests a viral strategy to evade senescence checkpoints, thereby extending the replicative lifespan of infected cells and promoting viral persistence and transformation.

Furthermore, the enrichment of HBV-related carcinogenic pathways in our analysis revealed overlapping disruptions in TFs such as NFE2L2 and the MEF2 family, suggesting that HPV may share oncogenic mechanisms with other DNA viruses. These findings point to convergent viral strategies that target host transcriptional networks to override tumor suppressive barriers and facilitate oncogenesis [62].

Finally, the unexpected enrichment of the axon guidance pathway suggests a non-canonical route by which HPV may exert oncogenic effects, specifically through the dysregulation of TFs that are traditionally associated with neurodevelopment and embryonic patterning. Among these, HOX, GLI3, and SOX family members emerged as significantly modulated in HPV-infected epithelial cells. These TFs are essential for maintaining cell polarity, directed migration, and spatial organization during development.

In the context of HPV-driven transformation, the alteration of these neurodevelopmental regulators may disrupt normal epithelial identity and structural integrity, EMT, and invasive phenotypes. HOX gene deregulation, for instance, is known to rewire transcriptional landscapes, promoting proliferation, motility, and loss of epithelial differentiation [63]. Likewise, GLI3, a critical mediator of the Hedgehog signaling pathway, regulates key decisions in cell cycle control and differentiation, and its aberrant activity has been implicated in multiple solid tumors, including those linked to viral oncogenesis [64]. The SOX family, including SOX2 and SOX17, further contributes to this phenotype by modulating stemness and cell plasticity, characteristics frequently enhanced in HPV-associated malignancies [65,66].

These findings suggest that HPV may choose evolutionarily conserved developmental pathways, originally tasked with embryonic tissue patterning, to orchestrate transcriptional reprogramming and microenvironment remodeling in favor of viral persistence and tumor progression.

**Figure 3:**
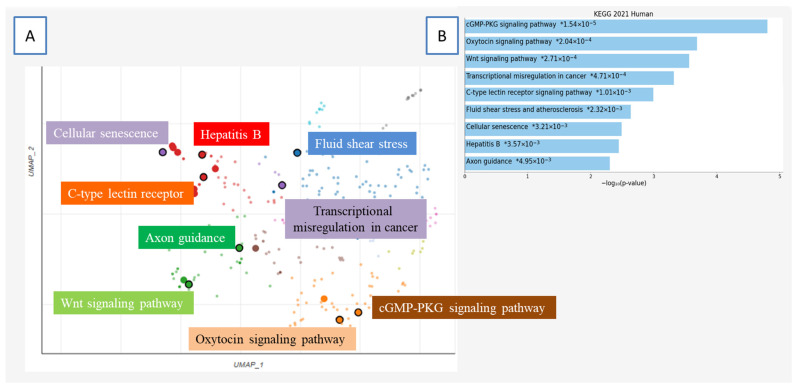
Functional enrichment analysis of genes associated with transcription factors, based on KEGG 2021 Human pathways. (**A**) UMAP (Uniform Manifold Approximation and Projection) visualization displays the clustering of genes based on pathway-level functional enrichment. Each cluster represents a significantly enriched KEGG pathway and is annotated with a distinct color-coded label. Prominent pathways include Wnt signaling, cellular senescence, axon guidance, and transcriptional misregulation in cancer, among others. This spatial distribution reflects the underlying transcriptional programs and their associated biological functions. (**B**) Bar chart showing the top significantly enriched KEGG pathways, ranked by statistical significance (−log_10_ (*p*-value)). Pathways such as cGMP-PKG signaling, oxytocin signaling, and Wnt signaling appear among the most highly enriched, underscoring their potential relevance in the transcriptional regulation associated with disease processes.

Taken together, these findings suggest that HPV-driven transformation is not limited to a few canonical pathways but instead involves a complex, multi-dimensional reprogramming of host transcriptional networks. The enrichment of previously unrecognized pathways and the identification of novel TF targets highlight the virus’s ability to modify a broad array of regulatory circuits that govern cell fate, differentiation, and structural integrity. This expanded understanding of HPV’s impact on host gene regulation opens new avenues for exploring how viral oncogenesis may operate through mechanisms far beyond those traditionally associated with HPV biology (Figure 3).

## 4. Discussion

High-risk HPV infections, particularly HPV-16 and HPV-18, should no longer be interpreted as isolated molecular accidents but rather as deliberate, systems-level subversions of the host cell’s regulatory architecture. The viral oncoproteins E6 and E7 are not merely pathogenic agents that inactivate cancer suppressors such as p53 and pRb; they act as central orchestrators of a broad transcriptional and epigenetic reprogramming effort that redefines the host cellular environment to favor viral persistence and replication.

Our findings extend the current understanding of E6/E7 functions by demonstrating that the mere presence of HPV is sufficient to induce widespread dysregulation of host transcriptional networks. Using computational analysis that integrates multiscale transcriptomic data with interaction-based inference, a complex cascade of molecular events has been described as potential consequences of viral infections and transcriptional misregulation of HPV viral DNA. These events could affect both transcription factors directly targeted by the virus and those indirectly influenced through downstream regulatory networks. These include master regulators of cell differentiation (e.g., HOX, CDX1), immune response (e.g., NFATC2/3/4, AIRE, DUX4), metabolic homeostasis and stress adaptation (e.g., CLOCK, NFE2L2), and chromatin remodeling (e.g., SMARCA1). Importantly, these associations are not stochastic; they converge on key processes such as immune evasion, EMT, genomic instability, and erosion of cellular identity.

This comprehensive network-based perspective marks a significant shift away from traditional reductionist models that focus on individual viral proteins or isolated pathways. Instead, the findings support viewing HPV as a systems-level disruptor that exploits the complex, emergent properties of transcriptional reprogramming within the host cell. A notable example is the consistent downregulation of DUX4, a double homeobox transcription factor that plays a critical role in zygotic genome activation and in the regulation of interferon-stimulated genes (ISGs). Its suppression suggests a key mechanism by which HPV may blunt innate immune responses during infection and transformation [67]. Similarly, the silencing of CDX1, a caudal-type homeobox gene critical for epithelial differentiation and Wnt repression, points to a broader loss of tissue identity [68]. These regulatory shifts reveal additional layers of HPV pathology that are rarely captured in conventional molecular studies.

Enrichment analysis using Enrichr further substantiates these insights. Beyond expected associations with canonical cancer pathways (e.g., Wnt signaling, transcriptional misregulation in cancer), our results revealed enrichment in unconventional signaling cascades. HPV appears to engage with a broad spectrum of host signaling cascades, including the C-type lectin receptor signaling pathway and the fluid shear stress response, both of which are not conventionally linked to oncogenesis. The CLR pathway plays a pivotal role in innate immune sensing, particularly in the detection of pathogens and the regulation of inflammatory responses, while the fluid shear stress response is primarily involved in mechanotransduction processes that influence vascular homeostasis and cytoskeletal organization [69].

HPV may use a variety of unconventional signaling systems to avoid immune surveillance, alter host cell stress responses, and create an environment that is conducive to viral persistence and cancerous development, according to the observed enrichment of these pathways. This highlights HPV’s capacity to exploit signaling networks beyond classical oncogenic routes, reinforcing a more complex and nuanced model of viral-driven transformation that extends well beyond traditional cell cycle deregulation and DNA repair inhibition.

A particularly striking feature of HPV pathogenesis is the mechanism of host shutoff, a phenomenon that appears paradoxical; while the virus relies on the host’s transcriptional and translational machinery to replicate, it simultaneously suppresses host gene expression to subvert immune responses and shift cellular resources toward viral replication. This selective repression is not indiscriminate but instead represents a finely tuned evolutionary adaptation, allowing HPV to degrade specific host mRNAs or prevent their translation while preserving or even enhancing the expression of cellular factors that facilitate its life cycle [70].

These mechanistic insights could have important therapeutic implications. The data challenge therapeutic approaches that focus solely on viral oncoproteins such as E6 and E7. Instead, a network-oriented strategy is here proposed, targeting the functional consequences of HPV-induced transcriptional reprogramming. Therapeutic interventions that aim to counteract host molecule dysregulations, e.g., modulate transcription factors (e.g., DUX4, CDX1), stabilize chromatin remodelers (e.g., SMARCA1), or disrupt oncogenic regulatory circuits, may provide lasting outcomes. Similarly, epigenetic modulators, or agents that restore the integrity of transcriptional networks, could potentially achieve greater efficacy than traditional single-target therapies.

More broadly, this study highlights the epistemological value of computational modeling in viral oncology. By simulating HPV–host interactions across multiple hierarchical regulatory levels, it becomes possible to characterize not only the direct targets of viral interference but also the underlying principles guiding these perturbations. Rather than acting as a random mutagenic agent, HPV functions as a strategic modulator within a co-evolutionary framework, systematically reshaping host transcriptional programs to create a permissive environment for viral persistence and replication. Decoding this regulatory logic uncovers actionable vulnerabilities and supports a shift toward systems-level therapeutic interventions based on an integrated understanding of host–virus dynamics.

## 5. Conclusions

This study positions HPV infection not as a linear molecular insult but as a multilayered event that simultaneously targets immune surveillance, cell identity, and chromatin dynamics. By employing a computational analytical pipeline, the HPV’s oncogenic potential arose not only from the well-characterized effects of E6 and E7 but also from an intricate connection with transcription factor activity and possible dysregulation.

The novelty of this approach lies in its ability to trace both direct and indirect interactions with viral DNA, revealing not just which TFs activity could be altered, but how their collective possible dysregulation could reshape the cellular system.

This underscores the importance of interpreting viral carcinogenesis through a network-based, integrative lens capable of capturing the full scope of molecular perturbations.

In this context, HPV is not only a biological adversary but also a force that confronts the host cell’s ability to resist various systems. It is now essential to comprehend this complexity to prevent, diagnose, and treat patients successfully. According to this viewpoint, future HPV research and innovative treatments should be built on the integration of systems biology, computational modeling, and transcriptional network analysis.

## Figures and Tables

**Table 2:** The table presents the top three DNA motifs discovered by MEME-ChIP in the analysis of HPV genomic sequences, along with their corresponding E-values and associated human transcription factors (TFs). The E-value indicates the statistical significance of each motif, with lower values reflecting higher confidence in the motif’s enrichment. The row color in the table for each motif corresponds to the color code used in Figure 1, specifically in the boxes containing the transcription factors (TFs) identified for each motif.

MEME-ChIP Motif (Sequence Logos)	E-Value	Human TFs
** 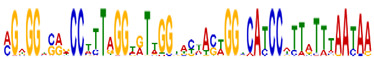 **	5.7 × 10^−128^	AIRE, ALX1, CLOCK, DBP, FOXC1, FOXJ3, FOXO4, FOXQ1, GLI3, HOXA1, HOXA9, HOXB13, HOXB7, HOXB8, LHX3, MAF, MEF2A, MEF2C
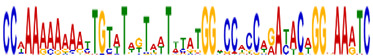	6.3 × 10^−127^	MEIS1, NFE2L2, NFATC2, NFATC3, NFATC4, NKX3-2, NKX6-1, NR1H3, OSR2, PBX2, SOX17, SRY
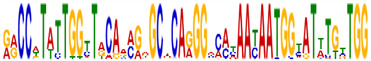	6.7 × 10^−127^	ZFP42, ZFP82, ZNF250, ZNF394, ZNF816, SMARCA1, DUX4, CDX1

## Data Availability

Publicly available datasets were analyzed in this study. These data can be found at: https://pave.niaid.nih.gov/explore/reference_genomes/human_genomes. All software used is open-source and freely accessible online.
